# Pediatric TMD: Associations with malocclusion, parafunctions, and symptoms: A cross-sectional study

**DOI:** 10.1097/MD.0000000000046115

**Published:** 2026-01-23

**Authors:** Yasemin Derya Fidancioğlu, Mutlu Güneş, Zeynep Yalçinkaya Kayhan

**Affiliations:** a Department of Pediatric Dentistry, Necmettin Erbakan University, Konya, Turkey; b Private Polyclinic, Istanbul, Türkiye; c Private Polyclinic, Konya, Turkey.

**Keywords:** cross-sectional study, early diagnosis, malocclusion, oral health-related quality of life, parafunctional habits, pediatric TMD, temporomandibular joint disorders

## Abstract

The aim of this study was to determine the prevalence of temporomandibular joint (TMJ) disorders and to investigate the role of malocclusion and harmful oral habits in their etiology among children. The rising prevalence of TMJ disorders in the pediatric population constitutes a significant clinical problem affecting their quality of life. A total of 242 patients aged 7 to 16 years who visited our clinic between March and September 2024 were included in this cross-sectional study. Patients were divided into 2 groups based on the presence or absence of TMJ disorders using the Fonseca questionnaire. Harmful oral habits were assessed with the Oral Behavior Checklist, and the impact on oral health with the Oral Health Impact Profile-14 questionnaires. Dental occlusion was determined according to Angle’s classification. Additionally, clinical findings such as joint sounds, tenderness, and mouth opening were recorded. Appropriate statistical tests were used for data analysis. TMJ disorders were detected in 63.2% of the patients. Statistical analyses revealed that the risk of TMJ disorder was 2.32 times higher in individuals with high scores for harmful oral habits (*P* < .001). The presence of Class II and Class III malocclusion increased this risk by 1.69 times (*P* = .005). In terms of gender, the incidence of TMJ disorders was 1.47 times higher in girls than in boys (*P* = .027). As the severity of the disorder increased, patients’ quality of life scores rose from 8.4 to 31.2 (*P* < .001). TMJ disorders were detected in 79.6% of patients who clenched their teeth at night. This study confirms that TMJ disorders are more prevalent than expected in the pediatric population. The findings indicate that harmful oral habits and malocclusion, particularly in Class II and Class III cases, are significant and modifiable risk factors for TMJ disorders. These results emphasize that careful assessment for these risk factors during routine pediatric dental examinations is a critical step for developing effective early diagnosis and intervention strategies.

## 1. Introduction

The temporomandibular joint (TMJ) is one of the most vital components of jaw function and possesses a complex anatomy. It primarily consists of the joint surfaces located between the mandibular condyle and the temporal bone’s mandibular fossa. The articular disc, a fibrocartilaginous structure that ensures harmony between these 2 bony components, regulates joint movements and facilitates load distribution. The joint capsule and ligaments (temporomandibular, stylomandibular, and sphenomandibular) support stability. Additionally, the associated masticatory muscles (masseter, temporalis, medial, and lateral pterygoids) are responsible for mandibular movements like opening, closing, protrusion, retrusion, and lateral excursions. The integrity and harmony of these structures are critical for the healthy execution of mechanical functions (chewing, speaking, and swallowing) and the prevention of pain and dysfunction.^[1,2]^

Temporomandibular disorders (TMD) are a complex clinical picture that affects not only the joint structures but also the functional integrity of muscles and surrounding tissues, manifesting with various symptoms. The symptoms of TMD are often multifaceted and directly impact patients’ daily lives. The most common signs include pain in the jaw joint, discomfort during chewing or at rest, limited or asymmetrical mouth opening, joint sounds such as clicking or crepitus, and jaw locking. Headaches, earaches, a feeling of pressure in the face, neck muscle tension, and even sleep disorders that reduce quality of life can also accompany the condition.^[3]^

Various therapeutic approaches are applied to alleviate these symptoms. For acute musculoskeletal discomfort associated with TMD, methods such as pharmacotherapy (indomethacin, diazepam, and paracetamol-orphenadrine citrate) and laser are utilized. Recent comparative clinical studies have shown that diode laser (940 nm) is superior to pharmacotherapy for TMD in controlling pain and improving mouth opening function. Patients treated with diode laser applications showed more lasting improvements both after treatment and during a 3-month follow-up, and fewer side effects were reported compared to the medication group.^[4]^

The occurrence of TMD in children and adolescents shows wide variation, with studies indicating that the condition affects 11% of children and 20% to 60% of adolescents. It is known that TMD in this age group has a multifactorial etiology, with malocclusion and oral parafunctions being important risk factors. Research indicates that TMD produces significant negative impacts on children’s quality of life related to oral health, which restricts their ability to perform daily tasks and damages their psychological health.^[1,2,5]^

Research studies about TMD occurrence and risk elements in children have produced inconsistent findings about how malocclusion affects TMD development. In particular, a valid protocol for the early diagnosis of TMD in the pediatric population has not yet been developed, and it is not sufficiently diagnosed in routine practice in this age group.^[5]^ Furthermore, there is no comprehensive study evaluating the relationship between malocclusion, parafunctional habits, and TMD symptoms in Turkish children using diagnostic criteria for temporomandibular disorders (DC/TMD) criteria, including quality of life.^[6,7]^ This study aims to fill a significant gap by thoroughly examining the effects of different types of malocclusion and oral parafunctions on TMD symptoms in children aged 7 to 16. Our findings will provide critical data for the development of early diagnosis and effective intervention strategies, contributing to the prevention of this condition that silently affects many children.

## 2. Methods

### 2.1. Study population

This cross-sectional study was conducted between March 2024 and September 2024 at the Paediatric Dentistry Clinic of Necmettin Erbakan University Faculty of Dentistry. Inclusion criteria were defined as being between 7 and 16 years of age, having no systemic disease, and obtaining parental consent. Patients undergoing orthodontic treatment, using space maintainers, any tooth with a crown having, teeth extracted due to caries or material loss, having craniofacial anomalies, having neurological or rheumatic diseases, having a history of scoliosis, having suffered any trauma to the head and neck region, and those with whom cooperation could not be established were excluded from the study. The Fonseca Anamnestic Index (FAI) defined temporomandibular dysfunction as patients who reached 20 points or more. The FAI scores divided TMD severity into 4 stages which started with no TMD (0–15 points) followed by mild TMD (20–40 points) and then moderate TMD (45–65 points) before reaching severe TMD (70–100 points). Parafunctional habits were operationalized as patients who performed at least one habit “sometimes” or more frequently on the Oral Habits Checklist.

### 2.2. Determination of sample size

The sample size of our study was determined by a power analysis performed using G*Power 3.1.9.7 software.^[8]^ Based on previous studies reporting a pediatric TMD prevalence of 11%^[9]^ and other studies reporting 27.2%,^[10]^ and based on studies reporting that more severe TMD symptoms occur in 5% to 9% and signs in up to 50% of the 10 to 15 age group,^[11]^ calculations were made with an expected prevalence of 25%. Using the parameters of type I error (α) 0.05, power (1-β) 0.80, and moderate effect size (Cohen d = 0.5), a minimum of 197 participants were calculated to be required for a single sample.

Taking into account subgroup analyses and possible losses (20% loss rate), a total of 242 participants were planned to be included in the study. The research design includes 60 to 80 participants in each of the 3 malocclusion groups (Angle Class I, II, and III) to achieve adequate statistical power for comparison. The power analysis for correlation studies showed that 242 participants would reach 85% power to detect a correlation coefficient of *R* = 0.30.

### 2.3. Study procedures

During the data collection process, parents were first informed about the study, their written consent was obtained, and demographic information was recorded. The Turkish version of the Fonseca Anamnestic Index was administered.^[12]^ This index consists of 10 questions, with each “yes” response scored as 10 points, “sometimes” as 5 points, and “no” as 0 points.

A 21-item form validated in Turkish was used for the Oral Habits Checklist, and each item was evaluated using a 5-point Likert scale (0–4 points).^[13]^ Oral Behavior Checklist (OBC) scores were grouped into 3 categories: low (<30 points), moderate (30–50 points), and high (>50 points). In the logistic regression analysis, an OBC > 40 points cutoff point was determined as a risk factor.

The Turkish version of the Oral Health Impact Profile-14 was used.^[14]^ Clinical examinations were performed by a single experienced pediatric dentist according to a standardized protocol in accordance with DC/TMD criteria.^[15]^ During the examination, angle classification, overjet and overbite measurements were recorded using a millimetric ruler, bilateral TMJ palpation was performed, and the maximum mouth opening and the distance between the upper and lower central incisors were measured.

The parental assessment questionnaire consisted of structured questions inquiring about the child’s nighttime teeth grinding, morning headaches, chewing difficulties, awareness of jaw joint sounds, history of trauma, TMJ treatment history, and psychiatric medication use.

### 2.4. Clinical assessment protocol

The dental unit chair served as the examination location where patients received their assessment while sitting upright. The Angle classification received its determination through evaluation of deciduous or permanent first molar occlusal relationships which resulted in Class I, II, or III classification. The measurements of overjet and overbite used a millimetric ruler to determine abnormal values when exceeding 6 mm and 5 mm, respectively. The TMJ examination started with stethoscope assessment of joint sounds while the patient performed mouth opening and closing movements to detect clicking or popping or crepitant sounds. Sensitivity in the TMJ and masticatory muscles (masseter, temporal) was assessed by bilateral palpation and recorded as present or absent.^[16]^ Maximum mouth opening was recorded in millimeters by measuring the distance between the incisal edges of the upper and lower central incisors when the patient was at maximum opening; values below 35 mm were considered limited. The presence of pain during function was assessed by asking the patient to perform chewing movements.

### 2.5. Statistical analysis

Data analysis was performed using Statistical Package for the Social Sciences v.26.0 (IBM Corp., Armonk) software. The Kolmogorov–Smirnov test evaluated the normal distribution pattern of continuous variables. The study presented mean (standard deviation) values for data that followed a normal distribution but used median (25th–75th percentile) to present data that did not follow a normal distribution pattern. The researchers presented categorical data through numerical values and percentage distributions.

The independent samples *t* test and one-way ANVA test identified Oral Health Impact Profile-14 (OHIP-14) score differences between TMD severity groups which were then compared to each OVA served to evaluate continuous variables that followed a normal distribution for intergroup assessment. The ANO other. The Mann–Whitney *U* test functioned as the statistical tool for examining variables which did not fulfillal-Wallis test because their data showed normal distribution patterns. The researchers used the Chi-square test to normal distribution criteria.

The researchers chose ANOVA for their 3-group analysis instead of the Krusk determine differences between categorical variables. The researchers used Pearson correlation analysis to assess the relationships between FAI score and OBC score and OHIP-14 score and age and maximum mouth opening measurements.

The researchers employed logistic regression to determine which elements lead to TMD development. In the regression model, the presence of TMD (FAI ≥ 20) was used as the dependent variable, and parafunctional habits (OBC > 40), presence of Angle Class II/III, gender, age group (12–16 years), and history of trauma were used as independent variables. The research findings presented odds ratio (OR) values and 95% confidence intervals (CI) together with *R*² model fit measurements. The study used *P* < .05 as its threshold for statistical significance in all analyses. List-wise deletion was applied for missing data, and the missing data rate was kept below 5%.

### 2.6. Ethical considerations

This study was approved by the Clinical Research Ethics Committee of Necmettin Erbakan University Faculty of Dentistry (2021/03-44). Written informed consent was obtained from all patients and their parents included in the study. The researchers anonymized and coded patient information to maintain confidentiality during analysis. The study team accessed encrypted computers and databases which contained the information through secure systems that protected data privacy. The research followed all principles established by the Helsinki Declaration.

## 3. Results

### 3.1. Participant characteristics

This cross-sectional study included a total of 242 children and adolescents who presented to the pediatric dentistry clinic between March and September 2024. The mean age was 11.4 ± 2.8 years; 118 participants (48.8%) were 7 to 11 years old and 124 (51.2%) were 12 to 16 years old. The cohort comprised 134 females (55.4%) and 108 males (44.6%). Participants were categorized by dental development stage into mixed dentition and permanent dentition groups (Table 1).

**Table 1:** Demographic and clinical characteristics of the participants.

Variables	n (%)	Mean (SD)
Demographic characteristics		
Age (years)		11.4 (2.8)
Mixed dentition (7–11 years)	118 (48.8)	
Permanent dentition (12–16 years)	124 (51.2)	
Gender		
Female	134 (55.4)	
Male	108 (44.6)	
*Orthodontic characteristics*		
Angle Classification		
Class I	98 (40.5)	
Class II	86 (35.5)	
Class III	58 (24.0)	
Crossbite	54 (22.3)	
Midline deviation	48 (19.8)	
Clinical TMD findings		
TMJ sounds (any)	128 (52.9)	
Clicking	68 (28.1)	
Popping	42 (17.4)	
Crepitation	18 (7.4)	
TMJ tenderness	94 (38.8)	
Masticatory muscle tenderness	86 (35.5)	
Pain during function	71 (29.3)	
Maximum mouth opening (mm)		42.3 (8.6)
Restricted (<35 mm)	38 (15.7)	
Tooth wear	76 (31.4)	

SD = standard deviation, TMD = temporomandibular disorders, TMJ = temporomandibular joint.

### 3.2. Clinical and occlusal findings

Orthodontic examination revealed Angle Class I malocclusion in 40.5% (n = 98), Class II in 35.5% (n = 86), and Class III in 24.0% (n = 58) of patients. Crossbite was observed in 22.3% (n = 54) and midline deviation in 19.8% (n = 48). On clinical assessment, 52.9% (n = 128) exhibited temporomandibular joint (TMJ) sounds, distributed as clicking in 28.1% (n = 68), popping in 17.4% (n = 42), and crepitation in 7.4% (n = 18). TMJ tenderness was present in 38.8% (n = 94), masticatory muscle tenderness in 35.5% (n = 86), and function-related pain in 29.3% (n = 71). The mean maximum mouth opening was 42.3 ± 8.6 mm, with 15.7% (n = 38) demonstrating a restricted opening < 35 mm. Tooth wear signs were recorded in 31.4% (n = 76) (Table 1).

### 3.3. Prevalence and severity of TMD

**Figure 1.**
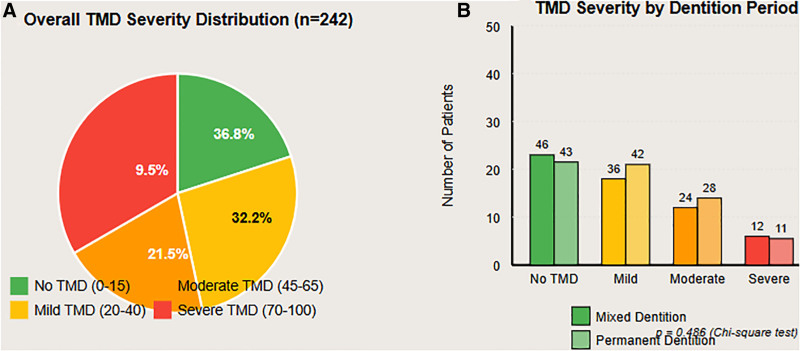
TMD prevalence and severity distribution: (A) overall distribution and (B) comparison between dentition periods. TMD = temporomandibular disorder; mixed = mixed dentition (7–11 years); permanent = permanent dentition (12–16 years).

Based on the FAI, 63.2% (n = 153) of the cohort met criteria for TMD, while 36.8% (n = 89) exhibited no TMD. TMD severity was distributed as mild in 32.2% (n = 78), moderate in 21.5% (n = 52), and severe in 9.5% (n = 23). The overall mean FAI score was 31.6 ± 23.4. By dentition stage, TMD prevalence was 61.0% in the mixed dentition and 65.3% in the permanent dentition, a difference not statistically significant (*P* = .486). These findings indicate a high frequency of TMD across pediatric age groups irrespective of dentition stage (Table 2; Fig. 1).

**Table 2:** Prevalence and severity of TMD by age groups.

TMD severity	Total n (%)	Mixed dentition n (%)	Permanent dentition n (%)	FAI score mean (SD)	OHIP-14 mean (SD)
No TMD (0–15 points)	89 (36.8)	46 (39.0)	43 (34.7)	8.2 (4.1)	8.4 (6.2)
Mild TMD (20–40 points)	78 (32.2)	36 (30.5)	42 (33.9)	28.5 (5.8)	14.6 (8.3)
Moderate TMD (45–65 points)	52 (21.5)	24 (20.3)	28 (22.6)	52.3 (6.2)	22.8 (9.7)
Severe TMD (70–100 points)	23 (9.5)	12 (10.2)	11 (8.9)	78.7 (7.9)	31.2 (11.4)
Total	242 (100)	118 (100)	124 (100)	31.6 (23.4)	15.8 (10.6)

Notes: *P*-value (age groups): 0.486 (Chi-square test); *P*-value (OHIP-14): <.001 (ANOVA test).

FAI = Fonseca Anamnestic Index, OHIP-14 = Oral Health Impact Profile-14, SD = standard deviation.

### 3.4. Oral health-related quality of life

OHIP-14 scores increased significantly with TMD severity (*P* < .001): children without TMD scored 8.4 ± 6.2, those with mild TMD 14.6 ± 8.3, moderate TMD 22.8 ± 9.7, and severe TMD 31.2 ± 11.4. This gradient underscores the substantial psychosocial and functional burden associated with escalating symptomatology. A strong positive correlation was observed between FAI and OHIP-14 (*R* = 0.72, *P* < .001) (Table 2; Fig. 1).

### 3.5. Parafunctional habits

The most frequently reported parafunctional behaviors were unilateral chewing and sleep-related clenching/grinding. The OBC mean score was 36.2 ± 17.4 in the mixed dentition and 40.9 ± 18.7 in the permanent dentition (*P* = .045). The mean OBC was 46.8 ± 16.4 in the TMD group and 24.5 ± 12.3 in the non-TMD group (*P* < .001). TMD prevalence rose markedly with higher OBC scores: 33.3% (26/78) in the low OBC group (<30) versus 92.6% (63/68) in the high OBC group (>50). FAI–OBC (*R* = 0.68, *P* < .001) and FAI–OHIP-14 (*R* = 0.72, *P* < .001) correlations further support the behavioral contribution to symptom burden (Table 3).

**Table 3:** Parafunctional habits by age groups and TMD presence.

Variables	Mixed dentition (n = 118)	Permanent dentition (n = 124)	With TMD (n = 153)	Without TMD (n = 89)	*P*-value
Habit scores*	Mean (SD)	Mean (SD)	Mean (SD)	Mean (SD)	
Unilateral chewing	2.08 (1.35)	2.28 (1.41)	2.64 (1.29)	1.38 (1.18)	<.001†
Night bruxism/clenching	2.04 (1.38)	2.35 (1.44)	2.76 (1.25)	1.24 (1.19)	<.001†
Nail biting	1.62 (1.43)	1.43 (1.39)	1.86 (1.42)	0.94 (1.20)	<.001†
Pen/object biting	1.56 (1.40)	1.40 (1.34)	1.78 (1.39)	0.96 (1.17)	<.001†
OBC total score	36.2 (17.4)	40.9 (18.7)	46.8 (16.4)	24.5 (12.3)	<.001†

OBC score groups	n (%)	n (%)	n (%)	n (%)	
Low (<30)	42 (35.6)	36 (29.0)	26 (17.0)	52 (58.4)	
Moderate (30–50)	46 (39.0)	50 (40.3)	64 (41.8)	32 (36.0)	<.001‡
High (>50)	30 (25.4)	38 (30.6)	63 (41.2)	5 (5.6)	

OBC = Oral Behavior Checklist, SD = standard deviation, TMD = temporomandibular disorders.

* 0 = never, 1 = rarely, 2 = sometimes, 3 = often, 4 = always.

† Mann–Whitney *U* test.

‡ Chi-square test; *P*-value (age groups OBC score): .045.

### 3.6. Multivariable analysis

**Figure 2.**
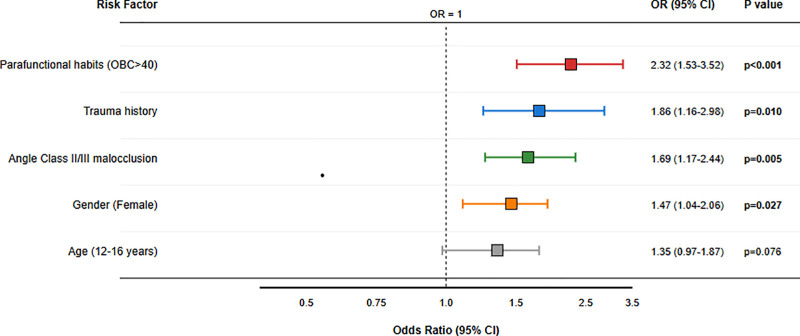
TMD risk factors: multivariate logistic regression analysis. TMD = temporomandibular disorder; OR = odds ratio; CI = confidence interval; OBC = Oral Behaviors Checklist.

In multivariable logistic regression adjusted for age, sex, occlusal class, parafunctional habits, and relevant clinical findings, independent predictors of TMD were identified as follows: OBC > 40: OR = 2.32, 95% CI: 1.53–3.52, *P* < .001; Angle Class II/III malocclusion: OR = 1.69, 95% CI: 1.17–2.44, *P* = .005; female sex: OR = 1.47, 95% CI: 1.04–2.06, *P* = .027. These estimates highlight the conjoint influence of behavioral and occlusal factors particularly parafunctional load on pediatric TMD risk (Table 4; Fig. 2).

**Table 4:** Risk factors for TMD (logistic regression analysis).

Risk factors	β	SE	OR	95% CI	*P*-value
Parafunctional habits (OBC > 40)	0.842	0.213	2.32	1.53–3.52	<.001
Angle Class II/III	0.524	0.186	1.69	1.17–2.44	.005
Gender (female)	0.382	0.172	1.47	1.04–2.06	.027
Age group (12–16 years)	0.298	0.168	1.35	0.97–1.87	.076
History of trauma	0.618	0.241	1.86	1.16–2.98	.010

*Notes*: Dependent variable: presence of TMD (FAI ≥ 20); model *R*² = 0.486, *P* < .001.

CI = confidence interval, FAI = Fonseca Anamnestic Index, OBC = Oral Behavior Checklist, OR = odds ratio, SE = standard error, β = regression coefficient.

### 3.7. Parental observations

Parent-reported behaviors and symptoms aligned closely with clinical findings. Nighttime clenching/grinding was associated with TMD in 79.6% (78/98) of affected children. The prevalence of this habit was 35.6% (42/118) during mixed dentition and 45.2% (56/124) during permanent dentition (*P* = .132). TMD was present in 80.6% (58/72) of children reporting morning headaches and 81.3% (52/64) of those with chewing difficulties. Among children with a history of trauma, TMD occurred in 73.7% (28/38) (Table 5).

**Table 5:** Parental observations by age groups and TMD presence.

Parental reports	Mixed dentition n (%)	Permanent dentition n (%)	With TMD n (%)	Without TMD n (%)	*P*-value
Night bruxism/clenching	42 (35.6)	56 (45.2)	78 (79.6)	20 (20.4)	<.001*
Morning headaches	30 (25.4)	42 (33.9)	58 (80.6)	14 (19.4)	<.001*
Chewing difficulties	26 (22.0)	38 (30.6)	52 (81.3)	12 (18.7)	<.001*
Awareness of TMJ sounds	38 (32.2)	48 (38.7)	68 (79.1)	18 (20.9)	<.001*
History of trauma	14 (11.9)	24 (19.4)	28 (73.7)	10 (26.3)	.018*

TMD = temporomandibular disorders, TMJ = temporomandibular joint.

* Chi-square test (for TMD presence); *P*-value (age groups for night bruxism): .132.

## 4. Discussion

### 4.1. General context and positioning of findings

This study was designed to provide a comprehensive assessment of the prevalence of TMD in children and adolescents and to explore the associated risk factors. Our findings revealed that 63.2% of participants presented with varying degrees of TMD, a figure substantially higher than many previously reported prevalence rates. This highlights TMD as a serious and underrecognized health burden in pediatric populations. Malocclusion, unilateral chewing, and particularly parafunctional habits such as nocturnal bruxism were strongly linked with increased risk, while female sex emerged as an independent predictor. Interestingly, no significant differences were observed between mixed and permanent dentition, suggesting that behavioral and occlusal loads outweigh chronological age or dental stage as determinants of TMD.

### 4.2. Prevalence and diagnostic variability

The prevalence of 63.2% reported here lies at the upper limit of the wide range (7–68%) identified in a recent meta-analysis by Alvear Miquilena et al.^[17]^ It also markedly exceeds the rates of 24.09% found by Macrì et al^[18]^ and 18.8% reported by Rentsch et al.^[19]^ Such discrepancies largely stem from diagnostic heterogeneity: we employed the FAI, a sensitive instrument that identifies self-reported symptoms often missed by purely clinical examinations. Our finding that prevalence was similar between mixed (61.0%) and permanent (65.3%) dentition stages (*P* = .486) aligns with prior research,^[18,19]^ reinforcing the view that developmental stage alone does not drive risk.

### 4.3. Gender differences: robust but multifactorial

We identified a 1.47-fold higher risk of TMD in girls (OR = 1.47, 95% CI: 1.04–2.06, *P* = .027). This observation supports earlier findings of increasing TMD symptoms among adolescent girls.^[6]^ Although some reports, such as that by Macrì et al, did not confirm gender-related differences,^[18]^ hormonal influences, psychosocial stress, and heightened pain sensitivity in females likely account for this disparity. This underscores the need for gender-sensitive approaches in pediatric TMD assessment.

### 4.4. Occlusal factors: mechanical susceptibility without exclusivity

Angle Class II/III malocclusions independently increased TMD risk by 1.69 times (OR = 1.69, 95% CI: 1.17–2.44, *P* = .005), consistent with reports linking these occlusal types to painful TMD.^[19]^ Our observed crossbite rate of 22.3% closely mirrors the 23.23% reported by Macrì et al.^[18]^ Moreover, longitudinal data by Myllymäki et al demonstrated that posterior crossbite increases the risk of TMJ sounds by 3.3-fold.^[20]^ Clinical signs further substantiate this association: TMJ sounds were present in 52.9% of children, crepitus in 7.4%, and restricted mouth opening (<35 mm) in 15.7%. These rates notably exceed those reported in some cohorts,^[18,21]^ emphasizing that occlusal imbalance and mechanical overload can potentiate TMD, though causation is clearly multifactorial.

### 4.5. Parafunctional habits: the dominant driver

Parafunctional behaviors exerted the strongest influence on TMD. Children with OBC scores > 40 had a 2.32-fold higher risk of developing TMD (OR = 2.32, 95% CI: 1.53–3.52, *P* < .001). Among those with high OBC scores (>50), prevalence reached 92.6%, while TMD was present in 79.6% of children with parent-reported nocturnal bruxism. These results strongly align with Torul et al, who reported a 4.24-fold increased risk associated with parafunctions,^[22]^ and with studies confirming bruxism prevalence between 13% to 49% in children.^[23,24]^ Correlation analyses demonstrated strong positive associations between FAI and OBC (*R* = 0.68) and between FAI and OHIP-14 (*R* = 0.72), confirming that the intensity of these behaviors directly scales symptom severity. Unilateral chewing and stress-related clenching may further accelerate this cycle, consistent with the anxiety-TMD link previously described.^[25]^

### 4.6. Quality of life impact

The OHIP-14 scores showed a striking gradient, rising from 8.4 ± 6.2 in children without TMD to 31.2 ± 11.4 in those with severe TMD (*P* < .001). This magnitude parallels findings in patients with TMJ osteoarthritis (scores 9.24–38.86)^[26]^ and in adolescents, where TMD increased the odds of impaired OHRQoL >4-fold.^[27,28]^ The observed correlation between FAI and OHIP-14 (*R* = 0.72) echoes the moderate-to-high correlations between OHRQoL and psychological distress in youth with TMD (rs = 0.53–0.61).^[29]^ Importantly, 80.6% of children reporting morning headaches and 81.3% of those with chewing difficulties were diagnosed with TMD, confirming that this disorder substantially disrupts daily function and psychosocial well-being. Trauma history also conferred a nearly 2-fold risk, further emphasizing the long-term burden of early physical insults.

### 4.7. Strengths, limitations, and future directions

The strengths of this study include the integration of clinical examination, validated indices (FAI, OBC, and OHIP-14), and multivariable regression, which allowed identification of independent predictors. Nonetheless, its cross-sectional design precludes causal inference, and the single-center clinical sample limits generalizability. Future research should adopt standardized DC/TMD protocols for children, include objective measures of bruxism (e.g., polysomnography), and expand to multicenter, longitudinal designs. We also highlight the potential role of postural and spinal factors, suggesting the necessity of a multidisciplinary framework involving dentists, orthodontists, physiotherapists, and mental health professionals. Indeed, a multidisciplinary study addressing these elements has already been initiated within our group.

## 5. Conclusion

This study demonstrates that temporomandibular disorders are far more prevalent in children than often assumed and represent a major public health concern. Three key insights emerge:

Behavioral load is the dominant determinant. High OBC scores and nocturnal bruxism identify a subgroup at markedly elevated risk, underscoring the importance of early behavioral interventions, stress management, and sleep-related habit counseling.

Occlusion contributes independently but synergistically. Class II/III malocclusions and crossbite significantly increase susceptibility; however, they act in concert with parafunctional and psychosocial factors, supporting a biopsychosocial model of pediatric TMD.

Quality of life deteriorates steeply with symptom severity. The OHIP-14 gradient and its strong correlation with FAI confirm that pediatric TMD is not a trivial condition, but one that severely impairs psychosocial functioning and daily activity.

*Clinical implication*: Routine TMJ screening, OBC-based behavioral assessment, and interdisciplinary referral should become standard components of pediatric dental care. Early identification and intervention are critical to prevent chronicity, mitigate disability, and safeguard the long-term quality of life of affected children.

## Acknowledgments

We thank all participants and their families for their cooperation.

## Author contributions

**Conceptualization:** Yasemin Derya Fidancioğlu, Zeynep Yalçinkaya Kayhan.

**Data curation:** Yasemin Derya Fidancioğlu, Mutlu Güneş.

**Formal analysis:** Yasemin Derya Fidancioğlu.

**Funding acquisition:** Yasemin Derya Fidancioğlu, Zeynep Yalçinkaya Kayhan.

**Investigation:** Yasemin Derya Fidancioğlu.

**Methodology:** Yasemin Derya Fidancioğlu, Mutlu Güneş.

**Project administration:** Yasemin Derya Fidancioğlu.

**Resources:** Yasemin Derya Fidancioğlu.

**Software:** Yasemin Derya Fidancioğlu.

**Supervision:** Yasemin Derya Fidancioğlu, Zeynep Yalçinkaya Kayhan.

**Validation:** Zeynep Yalçinkaya Kayhan.

**Visualization:** Zeynep Yalçinkaya Kayhan.

**Writing – original draft:** Yasemin Derya Fidancioğlu, Mutlu Güneş.

**Writing – review & editing:** Yasemin Derya Fidancioğlu, Mutlu Güneş.
